# Mortality in patients undergoing catheter-based therapy vs. anticoagulation alone for intermediate-high risk pulmonary embolism

**DOI:** 10.3389/fcvm.2026.1792855

**Published:** 2026-03-16

**Authors:** Marcus Brugger, Anna-Lena Schneider, Lorenz Mihatsch, Andre Kafka, Mark Lachmann, Katharina Bergmann, Jola Bresha, Ioana Ionac, Gregor S. Zimmermann, Christian Bradaric, Karl-Ludwig Laugwitz, Tareq Ibrahim, Arne M. Müller

**Affiliations:** 1 Department of Internal Medicine I, TUM School of Medicine and Health, TUM University Hospital, Technical University of Munich, Munich, Germany; 2 Department of Pediatrics, TUM School of Medicine and Health, TUM University Hospital, Technical University of Munich, Munich, Germany; 3 DZHK (German Center for Cardiovascular Research), Partner Site Munich Heart Alliance, Munich, Germany

**Keywords:** catheter-based mechanical thrombectomy, catheter-based thrombolysis, intermediate-high risk pulmonary embolism, pulmonary embolism, venous thromboembolism

## Abstract

**Introduction:**

Pulmonary embolism is a common and potentially fatal medical condition. Current guidelines recommend therapeutic anticoagulation for intermediate-high risk pulmonary embolism as a first-line strategy. However, it is unclear whether these patients may benefit from interventional treatments, such as catheter-based thrombectomy or catheter-based thrombolysis, compared to anticoagulation alone regarding mortality. Our aim was to gain insight into optimal treatment strategies of intermediate-high risk PE patients and to evaluate outcomes of different management strategies with regard to all-cause mortality.

**Methods:**

We retrospectively evaluated a total of 236 patients, 133 patients undergoing catheter-based thrombectomy or catheter-based thrombolysis (interventional group) and 103 patients receiving anticoagulation alone (conservative group) at our institution between 2003 and 2024. All patients were classified as intermediate-high risk. The primary endpoint was all-cause mortality.

**Results:**

The overall mortality for all patients with intermediate-high risk pulmonary embolism was 12.0% at 12 months. Depending on the treatment approach, all-cause mortality after 12 months occurred in 14 of 103 patients (14.0%) in the conservative group and in 14 of 133 patients (11.0%) in the interventional group which did not significantly differ (*p* = 0.150).

**Discussion:**

In patients with acute intermediate-high risk pulmonary embolism catheter-based treatment did not show a significant reduction in mortality within 12 months compared to anticoagulation alone.

## CENTRAL ILLUSTRATION

**Figure:**
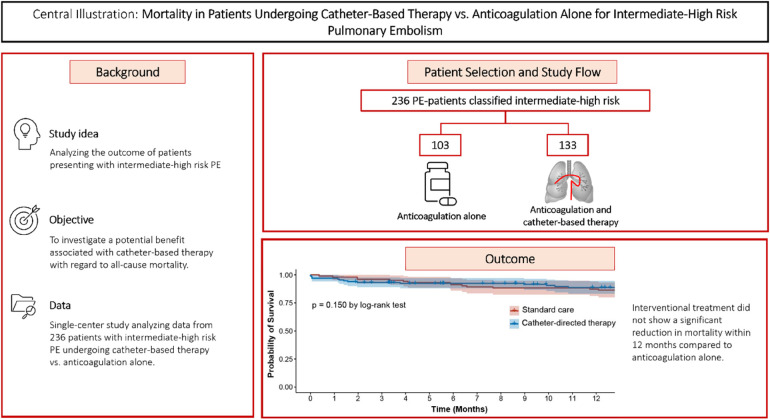
Graphical overview of background, patient selection, study flow and outcome of the study.

## Introduction

Acute pulmonary embolism (PE) is a major health concern and the third most common cardiovascular disease worldwide (1, 2). In 2020 the PE-related mortality rate was 12 per 100,000 population in the United States (3). In Europe, it is estimated that up to 370,000 deaths per year are associated with acute pulmonary embolism (4). The risk of developing pulmonary embolism increases with age. Due to demographic change, i.e., an ageing population, the number of patients will rise further over the next years (5). The treatment of low-risk and high-risk pulmonary embolism patients is clearly addressed in current guidelines. However, whether intermediate-high risk patients benefit from additional catheter-based strategies is yet unclear. Current international consensus guidelines recommend active thrombus removal strategies only in cases of acute decompensation or hemodynamic deterioration on anticoagulation treatment (6–8). Removal strategies include catheter-based thrombectomy or catheter-based thrombolysis. These novel techniques have received approval for clinical use based on prospective single-arm studies (9, 10) and postmarketing registries (11, 12). Still, the evidence for treatment with catheter-based procedures is sparse, since SEATTLE II, KNOCOUT PE, FLASH and FLARE mainly assessed imaging (RV/LV-ratio) and other surrogate endpoints including pulmonary artery pressure (PAP) rather than robust clinical outcomes e.g., mortality. Large-scale randomized trials supporting the use catheter-based therapies are underway (13, 14). Meanwhile, we aimed to retrospectively investigate whether patients with intermediate-high risk PE benefited from interventional treatment strategies (catheter-based thrombectomy or catheter-based thrombolysis) regarding mortality.

## Patients and methods

### Patients

We retrospectively evaluated all patients who underwent catheter-based thrombectomy or catheter-based thrombolysis at our institution between 2014 and 2024. Furthermore, we evaluated all patients with PE treated with anticoagulation alone between 2003 and 2013, since interventional treatment of acute PE was only started at our institution in 2014. This analysis included patients classified as intermediate-high risk only and provided written informed consent in case they received interventional treatment. Intermediate-high risk was defined according to the current *2019 ESC Guidelines for the diagnosis and management of acute pulmonary embolism* displaying hemodynamically stable patients with evidence of RV dysfunction and elevated cardiac high-sensitivity troponin T (hs-TnT) levels. The study was performed in accordance with the ethics committee of the Technical University of Munich (2023-560-S-NP) and adheres to the Declaration of Helsinki.

### Interventional treatment

Patients who underwent an interventional procedure either treated with catheter-based thrombectomy or catheter-based thrombolysis were included. The side of access for both procedures was the femoral vein. Pulmonary thrombectomy was performed using a mechanical thrombectomy system (FlowTriever®, Inari Medical, Irvine, California, United States). Catheter-based thrombolysis was provided by an ultrasound-assisted catheter-directed thrombolysis system, dissolving the thrombus locally (EKOS™ Endovascular System, Boston Scientific, Marlborough, Massachusetts, United States). In case of bilateral PE, the catheter was placed on the side of the most occluded pulmonary arteries. For thrombolysis, 5 mg of recombinant tissue plasminogen activator (r-tPA) was given as a bolus followed by 1 mg/h for 12 h. Intravenous heparin was administered during both procedures using a perfusor and the activated partial thromboplastin time (aPTT) aimed for was 60–80 s. The interventional protocol was left at the discretion of the treating interventionalist and therefore protocol deviations may have occurred. At discharge 42% of patients in this group received Rivaroxaban, 42% Apixaban, 4.5% low molecular weight or unfractionated heparin and 3.7% Phenprocoumon. Anticoagulation data at discharge was not available for 7.8% of patients.

### Conservative treatment

Patients treated by anticoagulation alone and who did not receive catheter-based treatment or rescue lysis served as a control group. Since catheter-based treatment strategies were introduced at our institution first after 2014, the control group was recruited between 2003 and 2013. Anticoagulation was performed according to guidelines and recommendations for anticoagulation in patients with PE. The choice of anticoagulation agent was left to the discretion of the treating physician. 77% of patients in this treatment group received Phenprocoumon, 5.7% Rivaroxaban and 12.5% low molecular weight or unfractionated heparin at discharge. For 5% of patients there was no anticoagulation data available at discharge.

### Database analysis

Patients with a history of central pulmonary embolism between 2003 and 2024 were identified using the center's electronic data management system and catheter laboratory database system. Central pulmonary embolism was defined by an objective proximal filling defect in at least one main pulmonary artery or pulmonary trunk detected by CTPA. Hemodynamically stable patients with objective evidence of right ventricular (RV) dysfunction based on CT scans and elevated cardiac high-sensitivity troponin T were included in the analysis (all classified intermediate-high risk).

### Primary endpoint

The primary endpoint was all-cause mortality following treatment for pulmonary embolism. Survival data were regularly obtained from the German Civil Registry, last requested in September 2024.

### Statistical analysis

Baseline demographic, clinical, and laboratory characteristics were summarized as mean ± standard deviation (SD), median, and interquartile range (IQR) for continuous variables and as counts and percentages for categorical variables. Differences between groups were tested using Kruskal–Wallis tests, Wilcoxon rank sum tests, Pearson's Chi-squared tests, and Fisher's exact tests as appropriate. Kaplan–Meier survival curves were constructed, and the log-rank test was used to compare the patients' survival between the two/three methods.

A multivariable Cox proportional hazards model was used to analyze the association between the intervention and overall survival, adjusting for active cancer. Hazard ratios (HR) with 95% confidence intervals (CI) were estimated. Model fit was assessed using the likelihood ratio test, and the concordance index.

The level of significance was set to 5%. Analysis was performed using R, Version 4.3.3 “Angel Food Cake” (The R Foundation for Statistical Computing, Vienna, Austria).

## Results

### Patients

Baseline characteristics of the patients are shown in Table 1. The mean age of the patients was 63 years; more patients were male (57%), with a mean BMI of 28.2 kg/m^2^. 21.0% of patients had a previous history of PE or deep vein thrombosis and only a small number of patients suffered from coagulopathy (4.7%). The mean troponin level was 0.08 ng/mL (high-sensitive troponin T, cut-off 0.014 ng/mL) and the average RV/LV-ratio was 1.83. A history of active cancer was present in 46 of 236 patients (19.0%). At the time of diagnosis, patients had normal hemoglobin and hematocrit levels. Cardiovascular comorbidities such as coronary artery disease were infrequently found in patients (9.7%). A total of 103 out of 236 patients were treated with anticoagulation alone, and 133 out of 236 patients received catheter-based treatment. 60 patients received catheter-based thrombectomy and 73 patients received catheter-based thrombolysis. Post-procedural bleeding complications at our center were very low. There was no major bleeding event after catheter-based thrombectomy (0 out of 60 patients) except minor bleeding at vascular access site, which was managed by compression. In 2 out of 73 patients who received catheter-based thrombolysis a major bleeding event occurred at the vascular access site without a need for further operational intervention. Compared to the PEERLESS study the most common source of bleeding in the catheter-based thrombectomy arm and catheter-based thrombolysis arm was the vascular access site (47.1% in LMBT vs. 62.5% in CDT of major bleeding events), without significant differences in major bleeding events in general (6.9% vs. 6.9%) (15).

**Table 1:** Baseline characteristics.

Characteristic	Overall, *N* = 236^a^	Conservative, *N* = 103^a^	Interventional, *N* = 133^a^	*p*-value^b^	SMD^c^
Age	63 ± 16 (66; 51–75)	63 ± 17 (66; 51–76)	62 ± 16 (66; 52–74)	0.517	0.061
Sex				0.694	0.052
female	102/236 (43%)	46/103 (45%)	56/133 (42%)		
male	134/236 (57%)	57/103 (55%)	77/133 (58%)		
BMI	28.2 ± 6.1 (26.9; 24.4–30.8)	28.0 ± 6.4 (26.6; 23.8–31.3)	28.3 ± 6.0 (27.0; 24.6–30.7)	0.617	0.048
Unknown	47	41	6		
Hypertension	100/236 (42%)	51/103 (50%)	49/133 (37%)	0.051	0.258
Any Lung Disease	33/236 (14%)	11/103 (11%)	22/133 (17%)	0.198	0.172
Coronary Artery Disease	23/236 (9.7%)	13/103 (13%)	10/133 (7.5%)	0.190	0.170
Any Cardiac Disease	42/236 (18%)	23/103 (22%)	19/133 (14%)	0.109	0.209
Coagulopathy	11/236 (4.7%)	5/103 (4.9%)	6/133 (4.5%)	1.00	0.016
GFR	77 ± 31 (75; 57–91)	74 ± 29 (72; 53–89)	79 ± 31 (75; 58–96)	0.219	0.167
History of DVT or PE	49/236 (21%)	21/103 (20%)	28/133 (21%)	0.901	0.016
Active Cancer	46/236 (19%)	27/103 (26%)	19/133 (14%)	0.022	0.300
Risk Classification					
Intermediate-high Risk	236/236 (100%)	103/103 (100%)	133/133 (100%)		-
Troponin	0.08 ± 0.09 (0.05; 0.02–0.10)	0.07 ± 0.09 (0.04; 0.02–0.08)	0.08 ± 0.09 (0.05; 0.03–0.11)	0.051	0.111
Hemoglobin	12.93 ± 2.40 (13.10; 11.30–14.60)	12.67 ± 2.33 (12.80; 11.10–14.65)	13.13 ± 2.45 (13.40; 11.60–14.60)	0.174	0.192
Hematocrit	38 ± 6 (39; 34–43)	38 ± 7 (38; 33–43)	38 ± 6 (39; 35–43)	0.323	0.001
Unknown	1	0	1		
RV/LV-Ratio	1.83 ± 0.63 (1.71; 1.37–2.13)	1.77 ± 0.53 (1.67; 1.31–2.09)	1.89 ± 0.70 (1.73; 1.41–2.17)	0.244	0.193

^a^ Mean ± SD (Median; IQR); *n*/*N* (%).

^b^ *p* values for comparison of patients with and without catheter-based treatment.

^c^ Standardized Mean Differences.

### Mortality during the first year

**Figure 1:**
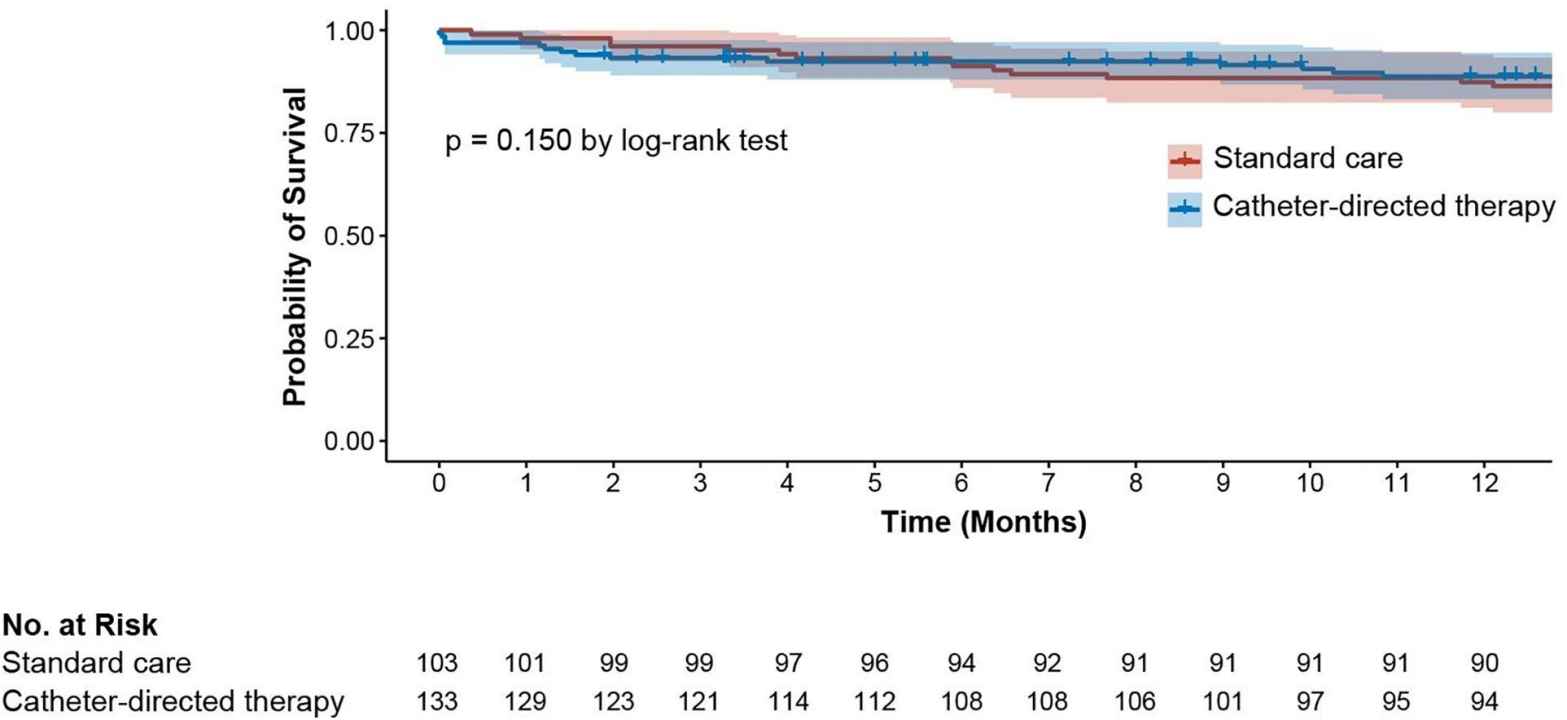
Kaplan-Meier probability of survival limited to 12 months.

After 30 days, 2 of 103 patients in the conservative group and 4 of 133 patients in the interventional group (1.9% vs. 3.0%, *p* = 0.699) met the primary clinical endpoint. At 6 months 6 of 103 patients died in the conservative group and 10 of 133 in the interventional group (5.8% vs. 7.5%, *p* = 0.608). After 1 year the mortality was 14.0% in the conservative group (14 of 103 patients) and 11.0% in the interventional group (14 of 133 patients, *p* = 0.150). Kaplan–Meier survival curves are shown in Figure 1. No difference in mortality was detected between catheter-based thrombolysis and catheter-based thrombectomy (*p* = 0.090).

### Long-term mortality

**Figure 2:**
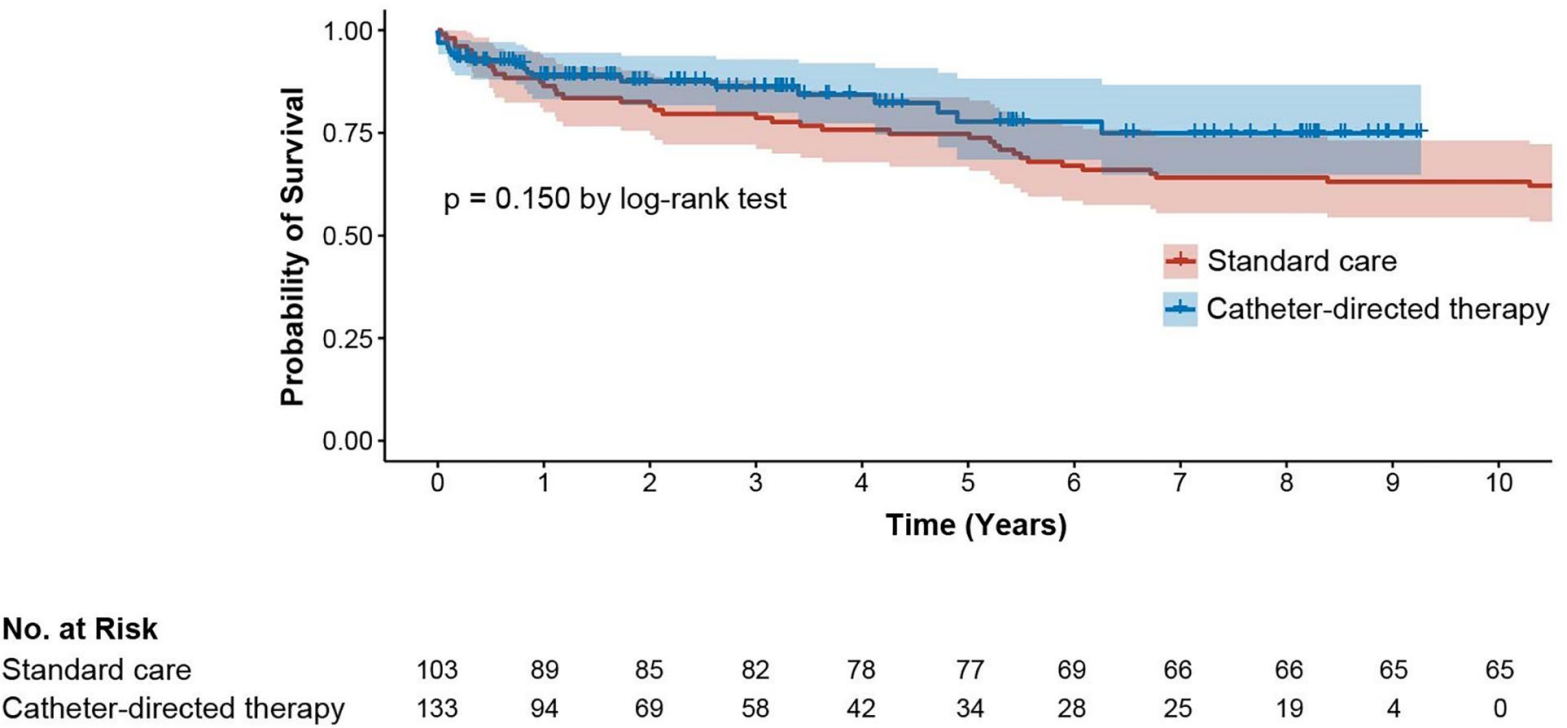
Long-term survival.

There was a trend towards reduced mortality in the interventional group compared to anticoagulation alone. However, this was not statistically different (*p* = 0.150) (Figure 2). Since active cancer was more prevalent, in a sensitivity Cox model adjusting for all baseline variables with moderate imbalance (active cancer, arterial hypertension, and any cardiac disease; |SMD| > 0.2), the intervention was not associated with improved survival [HR 1.11 (95% CI 0.62–1.98), *p* = 0.722]. Active cancer was strongly associated with mortality (HR 5.46, *p* < 0.001), and arterial hypertension showed a smaller association (HR 1.69, *p* = 0.040).

## Discussion

Intermediate-high risk pulmonary embolism (PE) is a serious medical condition, with patients being at high risk for rapid hemodynamic decompensation. In our analysis we have observed that catheter-based treatment for intermediate-high risk PE patients was not associated with a significant reduction in all-cause mortality after 1 year as compared to conservative treatment using anticoagulation alone.

Historically, therapy of intermediate-high risk PE patients with systemic thrombolysis in the PEITHO study led to a reduction in hemodynamic decompensation or circulatory collapse, but on the other hand, it increased the risk of major hemorrhage and stroke (16). Catheter-based thrombolysis, however, enables an application of thrombolytic agent directly into the thrombus, allowing a considerable dose reduction compared to systemic lysis and consequently lowering clinically relevant bleeding while reversing RV dilatation effectively (17–19). Since then, catheter-based procedures have gained popularity and, moreover, have been complemented by the development of new catheter-based thrombectomy systems. Nevertheless, clinical outcomes of catheter-based intervention strategies, either thrombolysis (9, 10) or thrombectomy (11, 12) for intermediate-high risk PE, remain unclear. Results of prospective single-arm studies using catheter-based thrombolysis (SEATTLE II, KNOCOUT PE) and post-marketing registries of thrombectomy (FLASH, FLARE) should be interpreted with caution to answer these questions, since a control arm with anticoagulation alone, which is the currently recommended standard in the treatment of intermediate-high risk PE, is lacking in these trials. Furthermore, they are subject to a selection bias due to the allowance of post-procedure consent and patients chosen to be good candidates for catheter-based treatment (11, 12). On the contrary, our analysis shows clinical real-life data including consecutive patients from different treatment eras with regard to the treatment strategy.

Strikingly, the prospective registry FLASH (catheter-based thrombectomy) showed a very low all-cause mortality of 0.8% at a 30-day follow-up (11), and similarly, the 30-day all-cause mortality of the KNOCOUT PE (catheter-based thrombolysis) prospective cohort was also low (1.0%) (12). In contrast, mortality in our study was remarkably higher, while the overall 30-day, 6- and 12-month mortality was 2.5%, 6.5%, and 12.0%, respectively. Similarly, the all-cause mortality with therapeutic anticoagulation as performed in the PEITHO study was 3.2% at 30 days (16).

Although catheter-based PE treatment is on the rise, it is noteworthy that the evidence for catheter-based strategies in randomized controlled trials is still sparse. The first randomized controlled trial to compare catheter-based strategies (PEERLESS) was published recently (15). In the PEERLESS study, catheter-based thrombectomy showed lower rates of clinical deterioration, escalation to bailout therapy, and intensive care unit utilization but detected no difference in mortality between individual catheter-based procedures at 30 days (0.4% for catheter-based thrombectomy and 0.8% for catheter-based thrombolysis) (15). In our study, the overall 30-day mortality was higher (30-day mortality 2.5%) than in the PEERLESS study. This might be associated with a higher rate of active cancer disease and a higher RV/LV ratio in our cohort. The high number of active cancer patients in our cohort could be due to our university hospital hosting a comprehensive cancer center and being 1 of 14 leading oncology centers in Germany.

Catheter-based treatment in our study was relatively well distributed, while 45% of patients received catheter-based thrombectomy and 55% received catheter-based thrombolysis (compared to PEERLESS: 50% thrombectomy, 50% thrombolysis). A further analysis showed no difference in mortality between catheter-based thrombolysis and catheter-based thrombectomy in our cohort (*p* = 0.090). However, the overall number of patients in our study is rather small. Larger randomized trials comparing different interventional approaches could be helpful to clarify potential advantages.

A randomized controlled trial comparing catheter-based thrombolysis vs. anticoagulation alone (standard therapy) with clinical endpoints is currently recruiting patients (HI-PEITHO) (13). The mortality rate of HI-PEITHO will be of great interest, since this study includes patients with severe cardiorespiratory distress.

Moreover, it is unknown whether acute catheter-based treatment can prevent life-threatening complications post-acute pulmonary embolism like chronic thromboembolic pulmonary hypertension (CTEPH), which occurs in 2.3% at 3 years (20). CTEPH, which may have an impact on long term mortality, could also be triggered by clots, which remain in smaller pulmonary arteries after catheter-based active thrombus removal strategies. The advantage of any catheter-based treatment of intermediate-high risk PE patients could be relevant for cancer-free patients with long life expectancy and should be investigated.

Further studies focusing on personalized medicine in the treatment of PE patients are needed to identify the optimal treatment, either a catheter-based strategy or anticoagulation alone for the individual patient. Hereby, the investigation of thrombus age and imaging could play a key role in acquiring a deeper understanding of intermediate high-risk PE.

### Limitations

Interpretation of the current findings should take potential limitations into account. Firstly, the study population is rather small to show a statistical effect on mortality. Secondly, two different time periods were analyzed for both groups. Therefore, a historical trend cannot be ruled out. It must be noted that the effect of the evolution of anticoagulation therapy is unknown. However, the improvement of efficiency and simplicity of anticoagulation therapy by using NOACs over the past years could have positively influenced the conservative treatment of PE (21). In our cohort 77% of patients received Phenprocoumon at discharge in the conservative group, while in the interventional group most patients were discharged with Apixaban and Rivaroxaban (42% each). Moreover, the improvement of ICU care over the past years and the launch of a Pulmonary Embolism Response Team (PERT) after introducing catheter-based treatment strategies at our institution in 2014 may have further influenced outcomes. Thirdly, we determined mortality based on the civil registry. The reason leading to death of the patients remains unclear since it was not recorded in this registry. It must be noted that about 20% of patients suffered from active cancer, which could be a substantial reason for death. Fourthly, residual confounding, particularly by unmeasured severity markers, contraindications, and secular trends, cannot be excluded. Therefore, results should be interpreted as associations rather than causal effects. Fifthly, we report on a single-center study and catheter-based thrombolysis protocols may vary between centers leading to a reduction in generalization of results. For example, other centers' protocols regarding recombinant tissue plasminogen activator (r-tPA) dose and duration of thrombolysis are both, lower in dosage and shorter in administration time compared to our protocol (17, 19). Sixthly, we decided to use CT scans to assess RV dysfunction only, since those were available for every single patient at the time of diagnosis and accessible for retrospective analysis. We did not include echo parameters, due to interobserver variability and missing data on echocardiography imaging at baseline and follow-up. Seventhly, data regarding patient compliance and therapy duration is not available. We assume that general practitioners will adhere to the current guidelines in the outpatient follow-up care of PE patients. Eighthly, data on bleeding complications at 1-year follow-up as well as recurrence rates of PE after 1 year are not available.

## Conclusion

In our study, interventional catheter-based treatment of patients with pulmonary embolism of intermediate-high risk was not superior to anticoagulation alone regarding mortality after 12 months.

### Impact on clinical practice

Since our analysis did not observe any significant difference in all-cause mortality rates, we suggest that intermediate high-risk patients can undergo catheter-based therapy with an overall low risk of mortality, although catheter-based treatment does not influence the standard of care for intermediate-risk PE regarding mortality. Independent, non-industry sponsored data (22) and industry-sponsored randomized controlled studies are underway and are needed to further justify the increased use of catheter-based treatments in intermediate-risk PE patients.

## Data Availability

The original contributions presented in the study are included in the article, further inquiries can be directed to the corresponding author.
